# Supplementary material for: Percutaneous kyphoplasty for osteoporotic vertebral compression fractures improves spino-pelvic alignment and global sagittal balance maximally in the thoracolumbar region
Source: PLoS One. 2020 Jan 30;15(1):e0228341. doi: 10.1371/journal.pone.0228341 (PMC6992186; doi:10.1371/journal.pone.0228341)
Supplement: Attachment — Submitted filename: Response to Reviewers.docx [file pone.0228341.s005.docx]

Dear Dr. Alejandro A. Espinoza Orías,

We are very grateful to you and the reviewers for giving us the opportunity to revise the manuscript entitled “Percutaneous Kyphoplasty for Osteoporotic Vertebral Compression Fractures Improves Spino-Pelvic Alignment and Global Sagittal Balance Maximally in the Thoracolumbar Region” (PONE-D-19-23099R1). We are heartened by the overall enthusiasm of you and the reviewers about the work, and appreciate the efforts to improve this work. We have studied the reviewers’ comments carefully and have made correction which we believe has led to an improved manuscript. These changes have been detailed in a point-by-point response as follows (Marked in red in the text):
**Reviewer's Responses to Questions：**
1. If the authors have adequately addressed your comments raised in a previous round of review and you feel that this manuscript is now acceptable for publication, you may indicate that here to bypass the “Comments to the Author” section, enter your conflict of interest statement in the “Confidential to Editor” section, and submit your "Accept" recommendation.
Reviewer #1: All comments have been addressed
Reviewer #4: (No Response)

**Response:** Thanks for the reviewer's affirmation of our revised manuscript. We revised the manuscript again according to the reviewer's comments, and strived to meet PLOS ONE’s publication criteria.

2. Is the manuscript technically sound, and do the data support the conclusions?
Reviewer #1: Yes
Reviewer #4: Partly

**Response:** We appreciate the reviewer’s comments. We asked professional statisticians to perform a statistical analysis of our data again, and added power analysis at the recommendation of reviewers.

3. Has the statistical analysis been performed appropriately and rigorously? 
Reviewer #1: I Don't Know
Reviewer #4: Yes

**Response:** Thanks to the reviewers for their affirmation of our statistical methods. All of our data statistics programs are consulted by statistical professionals, and it is believed that the statistical methods applied should be appropriate and rigorous**.**

4. Have the authors made all data underlying the findings in their manuscript fully available?
Reviewer #1: Yes
Reviewer #4: Yes

**Response:** Thanks for the two reviewers. All the data involved in our manuscript has been uploaded as an attachment during the submission.

5. Is the manuscript presented in an intelligible fashion and written in standard English?
Reviewer #1: Yes
Reviewer #4: No

**Response:** We invited native English speaking professionals to proofread our manuscript and revised the grammar and writing issues in the manuscript again. Please review it again and give us your valuable comments. Thank you!

6. Review Comments to the Author

**Reviewer #1:** Thank you for your work on this submission. I'm not able to perform statistical analysis which I leave to you and the other reviewer(s).

**Responses to Reviewer #1**

We would like to extend a special thank you for your helpful comments and recognition of our revised manuscript.

**Responses to Reviewer #4:**

We would like to extend a special thank you for your helpful comments and constructive criticism.
**1) Comment：**General comments: While the overall message is able to be conveyed, much of the wording is confusing and frequently grammatically incorrect. I encountered numerous errors in structure and grammar throughout the paper some of which have been highlighted in the specific comments section; however, there are many more throughout. Suggest review by another editor or thorough reworking to refine the syntax.

**Response:** Thanks for your valuable advice. We are very sorry for the many grammar and writing problems in the manuscript. We consulted native English speaking professionals to review our manuscript and made corresponding modifications to the grammar and writing issues in the text. Please review it again and give us your valuable comments. Thank you!

**2) Comment：**Was there a power analysis? Should include this in the statistical analysis section, especially given the small numbers for the non-TL groups, which may make the findings for these groups irrelevant. While this is addressed briefly in the drawbacks section, it should also be addressed in the discussion section and backed up by literature where applicable.
**Response:** We appreciate the reviewer’s comments. We have supplemented power analysis in the statistical analysis section (Line221-227) and discussed the results of power analysis in the discussion section (Line331-340). The power of the study was estimated using a post-hoc analysis with G*Power software (version 3.1.9.4, Franz Faul, Universität Kiel, Germany).With effect size of 0.8 and 0.05 level of statistical significance, the actual power of TL group was calculated to be 0.99. However, the powers of non-TL groups were ranged from 0.55 to 0.65. The reason is that osteoporotic vertebral compression fractures mainly occurred in the thoracolumbar region (T10-L2). Therefore, the number of patients in non-TL group was relatively small. And some of these patients also had fractures in the thoracolumbar region at the same time. In order to avoid confounding factors in the statistical analysis, we excluded patients with fractures in two or more different regions. Therefore, the number of patients in non-TL group was even smaller, which may have resulted in a higher statistical bias.

**3) Comment：**Lines 217 to 270 reads like a review of sagittal parameters and, while helpful, detracts from the overall findings of the study and is – to some degree – extraneous. If the purpose of the study is to describe an improvement in sagittal balance after PKP, then the discussion section should be focused on discussing these findings with appropriate references to support these findings (which the authors do in the latter half of the discussion section.) The discussion section needs to be trimmed down and focused on the relevant results.

**Response:** Thank you for your valuable comments. According to your advice, we have reduced some parts of the discussion.

In most cases, global sagittal balance is usually determined by measuring the SVA. Lines 243 to 271 explained why we chose SSA, TPA instead of SVA as the main parameters. We consider that this part should be kept in the manuscript. Thank you!

**4) Comment：**Specific Comments
Line 25: Reword this sentence – would not use contractions in a scientific paper. i.e. “However, the influence of kyphoplasty on spino-pelvic aligment and global sagittal balance when performed at specific treatment sites in the spine remains unclear.”
Line 37: Remove “of;” Sentence beginning
Line 72: Please combine with another paragraph – this sentence should not stand alone as its own paragraph.

**Response:** We are very sorry for these mistakes. We have made corresponding modifications according to the reviewer’s comments. Thank you!

**5) Comment：**

Line 46: improvement of what in the TL group? Needs to be reworded.

**Response:** Thank you for your suggestion. We have changed to “When PKP was performed, the improvement of sagittal balance parameters of TL group was the best in the three groups.”

**6) Comment：**
Line 69: Needs references to back up this claim, possibly naming specific examples.
**Response:** Thank you for your valuable advice. We have added the corresponding references to the manuscript, and taken spinal deformities as an example.

**7) Comment：**

Line 76: Sentence beginning “Whether the effect…” is confusingly worded.

**Response:** Thank you for your valuable comments. We have changed to “Whether there is a difference in the effect of thoracolumbar fracture site on sagittal balance has not yet been studied.”

**8) Comment：**

Line 101: Be specific regarding “hip and knee joint limitations.” I see that you addressed this in the previous reviewer comments, but it bears explaining in the text. Otherwise it remains confusing and vague.

**Response:** Thank you for your valuable comments. We have added an explanatory note to the manuscript to prevent readers from having the same doubts as you. We have changed to “Patients with hip and knee joint limitations (a history of hip and knee joint diseases, or abnormal hip and knee joint mobility in the medical records).”

9) **Comment：**

Line 224: Difficult to understand this sentence as written.

Line 238: Please provide a reference for this paragraph

**Response:** Considering that most of the first half of the discussion section is a review of sagittal parameters, we have deleted some paragraphs according to the reviewer’s comments. Line 224 and Line 238 have been removed already.

**10) Comment：**
Line 327: The sentence “After PKP, back pain…” is conjecture. The study did not measure back muscle strength and cannot claim improvement in strength as a reason for improvement in sagittal balance.
**Response:** Thank you for your valuable comments. At present, the influence of back muscle strength on sagittal balance is only our conjecture, which is not suitable for the discussion section. We have deleted it from the manuscript.

Special thanks to you for your good comments.

 We tried our best to improve the manuscript and made some changes in the manuscript. These changes will not influence the content and framework of the paper. And here we did not list the changes but marked in red in revised paper. We appreciate for Editors/Reviewers’ warm work earnestly, and hope that the correction will meet with approval.

Once again, thank you very much for your comments and suggestions.

Yours Sincerely,

Jianmin Sun

Department of spine surgery,

Shandong Provincial Hospital Affiliated to Shandong University, Jinan, P.R. China

No. 324 Jing Wu Road in Jinan city, 250021, P.R. China.

E-Mail: [sunjmsunjmsd@163.com](mailto:sunjmsunjmsd@163.com)
