# Supplementary material for: Percutaneous kyphoplasty for osteoporotic vertebral compression fractures improves spino-pelvic alignment and global sagittal balance maximally in the thoracolumbar region
Source: PLoS One. 2020 Jan 30;15(1):e0228341. doi: 10.1371/journal.pone.0228341 (PMC6992186; doi:10.1371/journal.pone.0228341)
Supplement: Attachment — Submitted filename: Response to Reviewers.docx [file pone.0228341.s006.docx]

Dear Dr. Alejandro A. Espinoza Orías,

We are very grateful to you and the reviewers for giving us the opportunity to revise the manuscript entitled “Percutaneous Kyphoplasty for Osteoporotic Vertebral Compression Fractures Improves Spino-Pelvic Alignment and Global Sagittal Balance Maximally in the Thoracolumbar Region” (PONE-D-19-23099R2). We are heartened by the overall enthusiasm of you and the reviewers about the work, and appreciate the efforts to improve this work. We have studied the reviewers’ comments carefully and have made correction which we believe has led to an improved manuscript. These changes have been detailed in a point-by-point response as follows (Marked in red in the text):
**Comment：**
The new paragraph on power analysis needs some improvement. It currently says:

"With effect size of 0.8 and 0.05 level of statistical significance, the actual power of TL group was calculated to be 0.99. Thus, our sample size of 71 patients was adequate, and the TL group was sufficiently powered to detect the effect of PKP on sagittal balance in patients with OVCF.
However, the powers of non-TL groups were ranged from 0.55 to 0.65. Based on these findings, the evidence is insufficient to investigate the parameters in non TL groups. Thus, additional studies with high-quality and large-scale are still warranted. "

It should say:

"With effect size of 0.8 and 0.05 level of statistical significance, the TL group (n=71) achieved a power of 0.99. Thus, the TL group was sufficiently powered to detect the effect of PKP on sagittal balance in patients with OVCF. However, power in the non-TL groups ranged from 0.55 to 0.65. Based on these findings, there is insufficient data to investigate the parameters in the non-TL groups. Thus, additional studies with proper large-scale cohorts are still warranted."

**Response:** We would like to extend a special thank you for your helpful comments and constructive criticism. According to your advice, we have made corresponding modifications in the manuscript. Thank you!

 We tried our best to improve the manuscript and made some changes in the manuscript. These changes will not influence the content and framework of the paper. And here we did not list the changes but marked in red in revised paper. We appreciate for Editors/Reviewers’ warm work earnestly, and hope that the correction will meet with approval.

Once again, thank you very much for your comments and suggestions.

Yours Sincerely,

Jianmin Sun

Department of spine surgery,

Shandong Provincial Hospital Affiliated to Shandong University, Jinan, P.R. China

No. 324 Jing Wu Road in Jinan city, 250021, P.R. China.

E-Mail: [sunjmsunjmsd@163.com](mailto:sunjmsunjmsd@163.com)
